# Evaluating the role of moonlight-darkness dynamics as proximate spawning cues in an *Acropora* coral

**DOI:** 10.1007/s00338-025-02618-9

**Published:** 2025-01-28

**Authors:** Rubén de la Torre Cerro, Elizabeth Beauchamp, Daisy Buzzoni, Jamie Craggs, Holly East, Alasdair Edwards, Yimnang Golbuu, Adriana Humanes, Liam Lachs, Helios Martínez, Aileen Mill, Eveline van der Steeg, Alex Ward, James R. Guest

**Affiliations:** 1 School of Natural and Environmental Sciences, Newcastle University, Newcastle Upon Tyne, UK; 2 Department of Geography and Environmental Sciences, Northumbria University, Newcastle Upon Tyne, UK; 3 University of Victoria, Victoria, BC Canada; 4 Horniman Museum and Gardens, London, UK; 5 Palau International Coral Reef Center, Koror, Palau

**Keywords:** Coral spawning, Moonlight, Phenological cues, Reproduction, Spawning Synchrony, Biological clocks

## Abstract

For sessile broadcast spawning marine invertebrates, such as corals, successful sexual reproduction depends on conspecifics spawning synchronously. The precise monthly, lunar, and diel timing and the extent of synchrony, i.e., proportion of population reproducing at the same time, are likely to play a key role in coral population recovery, persistence, and adaptation. Despite its importance, the mechanisms by which different environmental factors trigger corals to spawn on specific dates within the lunar cycle remain poorly understood. Periods of darkness post-sunset around full moon of the spawning month have been shown to induce spawning in merulinid corals, whereas for *Acropora,* moonlight is considered the main determinant driver of night of spawning. Here, we conducted two manipulative field experiments around full moon in Palau using the common table coral *Acropora* aff. *hyacinthus* to disentangle the role of moonlight and darkness post-sunset as proximate cues. Coral fragments were assigned to three treatments providing different post-sunset darkness conditions, versus control and procedural control fragments exposed to natural conditions. In contrast to previous studies on *Acropora*, we found that *Acropora* aff. *hyacinthus* can spawn synchronously in the absence of moonlight during the nights leading to spawning. Corals exposed to darkness post-sunset for at least two to three consecutive nights advanced their spawning compared to controls. This finding indicates that periods of darkness post-sunset can act as an inducer for spawning in *Acropora* as well as in merulinid corals, suggesting that this mechanism may be more widespread than previously thought.

**Supplementary Information:**

The online version contains supplementary material available at 10.1007/s00338-025-02618-9.

## Introduction

Lunar rhythmicity regulates many critical biological processes in marine invertebrates (Mercier and Hamel 2010; Tessmar-Raible et al. 2011). The association between lunar periodicity and timing of coral reproduction is well established (Stephenson et al. 1933) and both direct sensing of lunar irradiance and entraining of reproductive rhythms have been demonstrated by experimental moonlight manipulation (Jokiel et al. 1985; Gorbunov and Falkowski 2002). While the timing and degree of synchrony of coral spawning varies among taxa and locations (Baird et al. 2009; Harrison 2011), spawning often occurs during predictable periods of the lunar cycle. Various theories have sought to explain the ecological and evolutionary mechanisms behind synchronized reproductive behaviour. The most widely cited relate to predator satiation (Harrison et al. 1984), prevention of hybridization (Levitan et al. 2004), or gamete dilution and ageing (Levitan et al. 2011); however, the most parsimonious explanation is that higher levels of synchrony simply increase the chances of successful fertilization among conspecifics (Baird et al. 2009; Nozawa et al. 2015).

While it is well established that lunar rhythmicity influences coral reproductive behaviour, several other environmental parameters are also known to act as timing cues to determine the time of year (seasonal cycle), the date (lunar cycle), and the time of day (diel cycle) of coral spawning. These include rapid increases in sea surface temperature (SST) as the dominant proximate cue for the spawning month among Indo-Pacific *Acropora* (Keith et al. 2016), and maximum solar insolation, wind speed, tidal range, and rainfall associated with seasonal reproductive timing (Penland et al. 2004; van Woesik et al. 2006; Mendes and Woodley 2002; Wijayanti et al. 2019; Gouezo et al. 2020). Spawning can take place at any time within the lunar cycle, but most observations are centred around the full moon and can often be highly predictable (Baird et al. 2022; Bouwmeester et al. 2015; Lin and Nozawa 2017). The exact date of spawning is often associated with a specific period in the lunar cycle, suggesting an important role for lunar irradiance (Brady et al. 2016), although tidal changes could also conceivably play a role at an ultimate or proximate level (Kaiser and Neumann 2021). Lastly, the precise timing of spawning is strongly related to diel cycles with most observations occurring within a few hours after sunset, although a number of important reef-building corals spawn close to dusk (Brady et al. 2009), while a few species spawn during daytime (Bouwmeester et al. 2015; Eyal-Shaham et al. 2019; Lin and Nozawa 2017; Mangubhai et al. 2007). The precise timing of spawning can be manipulated by advancing the timing of sunset in mesocosms, demonstrating a direct role for diel light cycles in spawning induction (Craggs et al. 2017).

Endogenous clocks permit organisms to foresee changes in their environment and adjust their physiological and behavioural responses accordingly (Häfker et al. 2023). Corals have circalunar clocks entrained by environmental factors (e.g., moonlight) that allow them to synchronize spawning (Kaiser and Neumann 2021). While many environmental factors (such as temperature, salinity, turbidity, and cloud cover) change stochastically over short time scales (e.g., hourly or daily), changes in lunar irradiance over the lunar cycle occur predictably, making the lunar cycle a much more reliable pacemaker (Tessmar-Raible et al. 2011). Therefore, lunar irradiance is considered to be the most plausible regulatory mechanism for the date of coral spawning. Corals perceive changes in lunar irradiance thanks to photoreceptor proteins called cryptochromes, highly sensitive to the blue light spectrum, which enable them to track variations in moonlight intensity (Gorbunov and Falkowski 2002; Levy et al. 2007). These natural changes in moonlight photoperiod over the lunar month regulate the expression of a pool of genes involved in the spawning process (Brady et al. 2016; Kaniewska et al. 2015; Levy et al. 2007; Rosenberg et al. 2017; Rosenberg et al. 2017; Wuitchik et al. 2019). Thus, evidence until now has led to the acceptance of moonlight as the primary cue determining day of spawning, with changes in moonlight intensity as a trigger (Davies et al. 2014). This model has been supported by research showing that disruption to natural moonlight patterns, such as that of artificial light pollution at night (ALAN) can alter timing of coral spawning (Davies et al. 2023), and possibly suppress spawning (Ayalon et al. 2021; Kaniewska et al. 2015; Lin et al. 2021). Further, the absence of moonlight has also been demonstrated to have a suppressive effect on spawning, particularly for corals in the genus *Acropora* (Kaniewska et al. 2015).

Recent research on merulinid corals has questioned the role of lunar irradiance as the factor inducing spawning. Lin et al. (2021) demonstrated a suppressive effect of moonlight and artificial light on the spawning of *Dipsastraea speciosa,* whereas a darkness period after sunset triggered it, acting as a proximate cue. These results suggest that a darkness interval between sunset and moonrise could trigger spawning; however, it is not known if this model applies to other coral species. For *Acropora* and Merulinidae, a combination of cumulative moonlight and temperature signals has been recently suggested as cues for spawning (Komoto et al. 2023). Through their proposed coincidence model of cumulative temperature and moonlight cues, Komoto et al. (2023) suggested that *Acropora* corals are more sensitive to moonlight than Merulinidae corals and thus respond more readily to lunar irradiance cues. Further, their model proposed the existence of a gating period, a specific time window in which *Acropora* are most influenced by moonlight, taking place after midnight. Thus, presence or absence of moonlight during the proposed gating period would act as a cue to initiate essential processes for spawning. However, it remains unknown whether moonlight or the darkness period post-sunset are the ultimate cues that induce spawning to take place on a given night of the lunar cycle for *Acropora*.

*Acropora* corals have been documented to spawn across a broad range of dates within the lunar cycle (Baird et al. 2021), often presenting marked species-specific degree of synchrony within local assemblages, spawning over one or a few consecutive nights (Bouwmeester et al. 2015; Lin and Nozawa 2017; Sakai et al. 2020). Among *Acropora* species, spawning dates occur over a gradient of moonlight and darkness conditions, ranging from full sky illumination (e.g., moonrise before sunset) to increasing intervals of darkness after sunset (e.g., when moonrise occurs progressively later each day after sunset). The former typically happens during the nights preceding the full moon, while the latter occurs towards last quarter moon, as the moon rises approximately 30–70 min later each night. Recent research shows how lunar rhythms modulate changes in gene expression in *Acropora* corals over different phases on synodic cycles (Brady et al. 2016; Oldach et al. 2017) and highlights their ability to sense changes in night illumination (Gorbunov and Falkowski 2002; Brady et al. 2016).

Despite recent advances in our understanding of coral spawning, the exact mechanism that induces *Acropora* corals to spawn on a particular night within the lunar cycle remains unknown. The following specific questions remain unanswered: (1) Is there variability in spawning night relative to the full moon in split spawning populations? (2) If moonlight is key to induce spawning, why do *Acropora* spawn in a range of lit and unlit conditions at night over the lunar cycle? (3) Could a period of darkness be the ultimate cue to induce *Acropora* corals to spawn synchronously on a specific night of the lunar cycle, as demonstrated in some merulinid corals? The aim of the present study was to determine if a darkness period post-sunset might serve as a cue for spawning timing in an *Acropora* coral, as the current proposed model for merulinid corals (Lin et al. 2021) and to investigate the effect of various moonlight manipulation treatments on the spawning timing and synchrony of the study species. To do this, we used an in situ field experiment to manipulate the amount of moonlight and darkness post-sunset over two consecutive lunar cycles for a common *Acropora* species in Palau. We tested whether a darkness post-sunset period provides a cue for the night of gamete release in *Acropora* and if the absence of moonlight could alter or suppress spawning.

## Materials and methods

### Study site and species

Field experiments were conducted in the Republic of Palau (7° 33′ N, 134° 0.46′ E) during March and April 2022. *Acropora* aff. *hyacinthus*, a hermaphroditic broadcasting scleractinian coral that spawns in March and April in Palau (Baird et al. 2021; Gouezo et al. 2020) was selected for the study. The “aff.” qualifier refers to specimens that have morphological affinities with a nominal species (Cowman et al. 2020). Colonies were chosen based on visual identification of colony morphology corresponding to the descriptions of the nominal species (fragment images are available in Supplementary materials S1). Corals were collected from a shallow exposed reef east of Koror (Mascherchur reef 7° 17′ 29.3″ N, 134° 31′ 8.0″ E) prior to the March and April full moons by divers using a hammer and chisel. Gravid colonies were identified by removing up to three branches and noting the presence of pigmented oocytes following Baird and Marshall (2002). A total of five fragments (> 10 cm diameter) were excised from 10 randomly selected colonies in both months. The experiments were carried out at a sheltered reef site adjacent to the Palau International Coral Reef Center (PICRC), approximately 8 km from the donor site.

Fragments were tagged and transported in coolers to PICRC (travel time ~ 30 min). Initially, colony fragments were maintained for 24 h in tanks (~ 760L) with a flow through system of 50 µm filtered seawater and two water pumps (Hydor Koralia Nano Circulation Pump/Powerhead and one Taam Rio + 800 Powerhead) to promote water flow. After checking for damage and signs of partial mortality, healthy fragments were transferred to the experimental site adjacent to PICRC two days prior to the commencement of experiments. Corals were attached with cable ties to sheets of plastic egg crate raised approx. 40 cm above the bottom at a depth of 2.5–3 m, situated > 100 m away from any artificial light for the duration of each experiment. SST range at the experimental site was similar to that of the donor reef during mid-March–end of April (28.78–30.9 and 28.5–31 °C, respectively), differences in hourly SST between experimental and donor sites ranged from + 1.8 °C to − 0.34 °C (supplementary materials, Fig. S2).

### Experimental design

The date on which experiments commenced in each month was determined based on the local timing of moonrise relative to sunset and known spawning timings of the study species. The lunar photoperiod on the days close to full moon (FM) date is very similar in each lunation. A few nights prior full moon there is almost continuous illumination in the evening sky, since the moon rises before sunset and moonset occurs progressively closer to dawn. Then, on the days after full moon, moonrise time takes place progressively later after sunset (Fig S4). This creates an increasing period of darkness post-sunset towards the first quarter moon, that ranges from a few minutes the day after FM to around 2 h of darkness post-sunset three days after FM. *Acropora* corals in the Indo-Pacific have been documented to spawn over a wide range of dates within the lunar cycle, from 15 days before to 14 days after the full moon (Baird et al. 2021). However, most observations (56% of records) occur between 3 and 6 days after the full moon (Baird et al. 2021, 2022; Sakai et al. 2020). In Palau, *Acropora* corals tend to spawn between March and April, split spawning is common for some taxa in Palauan reefs and its occurrence seems to be associated with March FM falling in the middle or later part of the month (Gouezo et al. 2020). Colonies that spawn on consecutive full moons tend to do so a few days after the full moon in March, and very close to, or a few days before FM night in April (Gouezo et al. 2020). Thus, the March spawning experiment commenced 2 days before FM night (March 18th), until 7 days after FM, whereas the April experiment started 5 days prior FM night (April 17th).

### Moonlight blocking treatments

Five fragments per donor colony (*N* = 10) were randomly assigned so that one fragment from each genotype was present in each of the five treatments, following a stratified random design (supplementary materials 3). Three blockage treatments were used to manipulate moonlight conditions as follows: Full Blockage (FB), in which moonlight was blocked over the entire duration of the experiment; Early Blockage (EB), in which moonlight was blocked for the first three days of the experiment, after which fragments were exposed to environmental moonlight conditions for the rest of the experiment; and Late Blockage (LB), in which fragments were exposed to environmental moonlight conditions during the first 3 days of the experiment, after which moonlight was blocked until the end of the experiment.

**Fig. 1:**
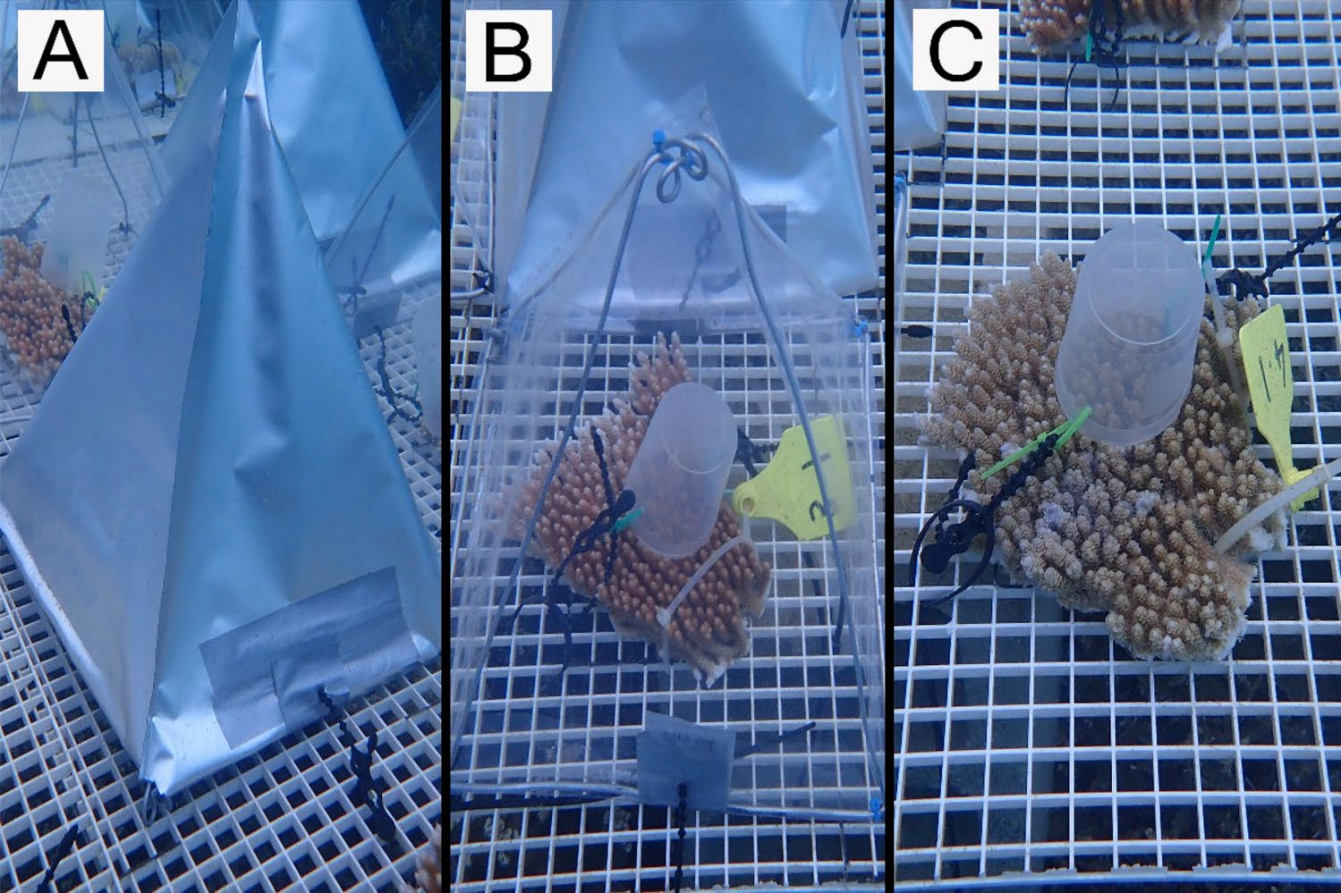
Example of devices used in the different treatments to manipulate moonlight exposure. **A** opaque cover to prevent incident light reaching the coral fragment. **B** Transparent cover to allow incident light to reach the coral fragment. **C** Control with no cover, but with a collection cup. All fragments had plastic cups attached to collect gamete bundles if colonies spawned. Opaque covers were used on treatments full, early, and late blockage. Transparent covers were used in procedural controls, early, and late blockage treatments

Experimental devices were made using pyramid-like metal frames, with a base of approximately 25 cm × 25 cm, combined with aluminium foil bags (Fig. 1A) or transparent plastic bags (Fig. 1B) fixed to the frames using a heat sealer. Moonlight was blocked by covering fragments with individual opaque covers (Fig. 1A). Two control treatments consisted of fragments with no covering throughout the duration of the experiment (Control) and fragments covered with transparent covers, as procedural controls (Procedural Control) (Fig. 1C, B respectively). All covers were placed on their corresponding fragments by attaching them to the egg crate with cable ties. The uncovered bottom facilitated water exchange in the devices. Covers were attached every evening at sunset (18:15 h) and removed each morning at sunrise (06:00 h). After finishing the March spawning experiment, experimental fragments were returned to the donor reef and reattached to the substratum. Fifty new fragments, five from each of ten new colonies, were collected from the donor reef prior to commencing the second experiment in April, which consisted of the same five treatments (Supplementary materials S3, Tables S1 and S2).

### Spawning monitoring

Collection cups (120 mL) were attached to each individual fragment with cable ties to serve as gamete bundle traps (Fig. 1B, C). After carefully removing covers (where present), collection cups were examined each morning, and spawning status was recorded in one of three categories (major spawning if ≥ 100 gamete bundles; minor spawning if < 100 gamete bundles; and no spawning if 0 gametes). Health status of fragments was also monitored each morning according to five categories; healthy, partially bleached, bleached, partial mortality, or dead. At the end of both experiments, branches were excised from each fragment to determine if mature oocytes were visible, and their reproductive stage was recorded in two categories; empty, if no eggs were found, or mature, if pigmented eggs were still present by the end of the experiments. Spawning monitoring took place only within the experimental site, visits to the donor reef to monitor spawning were not possible due to logistics; however, it would be valuable for future studies to also acquire such data.

### Data analysis

The effect of treatments (five categorical levels, fixed effect) and lunar night (continuous variable, fixed effect) on *Acropora* spawning (binary response) were tested independently for each experiment using generalized linear mixed-effect models (GLMMs) with a binomial error distribution. A random intercept for each donor colony (Colony_ID) was considered to account for other sources of variation in the fragment’s response, such as genotype effects. We determined spawning night as the probability of two events, namely a fragment spawned on a particular night (1), or fragment had not spawned on a particular night (0). For our analyses, spawning night was considered as the first night a fragment had a major spawning event, when a fragment had more than one major spawning events, the first major spawning event was used. There were two reasons for us to take this approach; firstly, to avoid repeated measures, secondly, multiple major spawning events only took place for a few fragments, and, in such cases, it always happened over two consecutive nights. Therefore, averaging across two consecutive dates was not possible. The same approach was taken in the case that a fragment did not have a major spawning event but had more than one minor spawning events, the first night in which spawning was observed was used for the analyses. Tukey’s honestly significant difference tests for pairwise comparisons were performed among experimental treatments. All analyses were carried out in R software (version 4.2.1) and the packages nlme4 (version 1.1-30), car (version 3.1-10), and glmmTMB (version 1.1.4).

## Results

### Effect of moonlight blocking treatments, March spawning

**Fig. 2:**
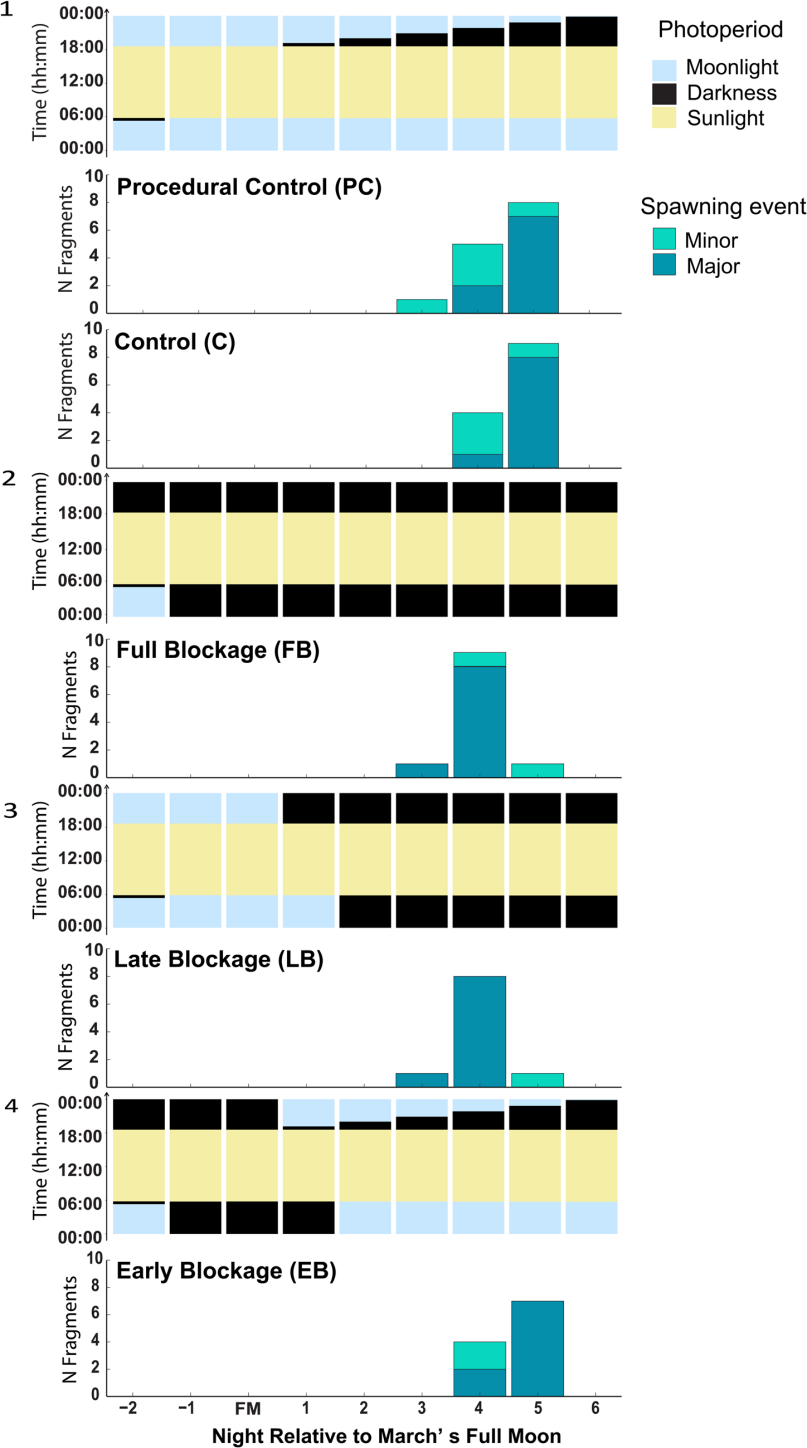
Spawning of *Acropora*. aff. *hyacinthus* fragments in the March experiment. Panels 1–4 illustrate the daily light-darkness profiles corresponding to each treatment. For each day, yellow, pale blue and black bars in the light regime panels indicate periods of sunlight, moonlight, and darkness, respectively, experienced by the colony fragments in each treatment. (1) Environmental conditions, treatments Control (C) and Procedural Control (PC), based on sun and moon rise and set times in Koror, in which no moonlight-darkness manipulations were performed. (2) Full Blockage (FB) treatment, moonlight was blocked post-sunset from 2 days prior to the full moon day until the end of experiment. (3) Late Blockage (LB) treatment, coral fragments were exposed to environmental moonlight conditions until the full moon day then given total darkness post-sunset from the day after full moon. (4) Early Blockage (EB) treatment, corals were exposed to total darkness post-sunset the first three nights then given environmental conditions from a day after the full moon. Bar graphs underneath light regime panels indicate the spawning pattern on each treatment. Dark teal bars indicate major spawning, light teal bars indicate minor spawning

In March, most fragments (92%) underwent major spawning although the night of spawning varied among treatments (Fig. 2). Most fragments in control and procedural control treatments spawned together five nights after FM, with one and two fragments, respectively, spawning on the night before FM. The spawning window for these fragments was within the range of known spawning dates (+ 3 to + 6 days after FM) for *Acropora* in Palau (Table 1). For fragments where moonlight had been blocked throughout the experiment (Full blockage, FB) or blocked for 3 days after the start of the experiment (Late Blockage, LB), spawning predominantly (_**~**_90%) occurred one night earlier (i.e., 4 days after FM) compared to the control treatments. However, fragments for which moonlight was blocked at the start of the experiment and later exposed to moonlight (Early Blockage, EB) behaved similarly to those in the control treatments.

**Table 1:** Spawning records of *Acropora hyacinthus* (*Acropora* aff. *hyacinthus*) in Palau from this study and a combination of published and unpublished data

Species	Location	Year	Date	SNRNFM	Time	Observation	References
*Acropora hyacinthus*	UCH	2009	15/04/2009	+ 5	20:45	ex situ	Boch and Morse (2012)
*Acropora hyacinthus*	UCH	2013	30/03/2013	+ 3	–	in situ	Baird et al. (2021)
*Acropora hyacinthus*	UCH	2013	31/03/2013	+ 4	–	in situ	Baird et al. (2021)
*Acropora hyacinthus*	UCH	2017	09/04/2017	− 2	–	in situ	Gouezo et al. (2020)
*Acropora hyacinthus*	UCH	2018	02/04/2018	+ 2	–	in situ	Gouezo et al. (2020)
*Acropora hyacinthus*	ULO	2020	12/04/2020	+ 4	19:00–21:00	in situ	Gouezo (unpublished)
*Acropora* aff.* hyacinthus*	PICRC	2021	02/04/2021	+ 4	20:30	ex situ	van der Steeg (unpublished)
*Acropora* aff.* hyacinthus*	PICRC	2021	05/04/2021	+ 7	20:45	ex situ	van der Steeg (unpublished)
*Acropora* aff.* hyacinthus*	ULO	2021	06/04/2021	+ 8	20:30	ex situ	van der Steeg (unpublished)
*Acropora* aff.* hyacinthus*	UCH	2022	19, 20/03/2022*	+ 1/+ 2	–	Inferred*	Ricardo et al. (unpublished)
*Acropora* aff.* hyacinthus*	MSC	2022	22/03/2022	+ 4	–	in situ	This study
*Acropora* aff.* hyacinthus*	UCH-NB	2022	22/03/2022	+ 4	21:15	in situ	Ricardo et al. (unpublished)
*Acropora* aff.* hyacinthus*	MSC	2022	23/03/2022	+ 5	–	in situ	This study
*Acropora* aff.* hyacinthus*	MSC	2022	15/04/2022	− 2	–	in situ	This study

Species refers to *Acropora hyacinthus* or morphologically similar (aff) species. Location refers to reefs where at least one colony of this species was observed to spawn. Location codes refer to; UCH = Uchelbeluu, ULO = Ulong, MSC = Mascherchur, PICRC = PICRC research station, UCH-NB = Uchelbeluu (Donor reef), relocated and spawned in situ at Ngermid Bay. Year and date columns refer to the year and night of spawning, respectively. SNRNFM refers to the night of spawning in days, before (+) or after (−) the closest full moon to each spawning record. Time refers to the start time of spawning, (–) denotes no spawning time was recorded. Observation: “ex situ” when spawning took place out of the donor reef, in a controlled set up, “in situ” refers to observations in the reef, either donor or receptor. Inferred* illustrates spawning was not observed but was documented through gamete sampling. In the one specific case, it refers to an observation in which colonies presented pigmented gametes on March 19th, then gametes were absent on March 21st. Reference refers to the published or unpublished source of the compiled spawning records

**Fig. 3:**
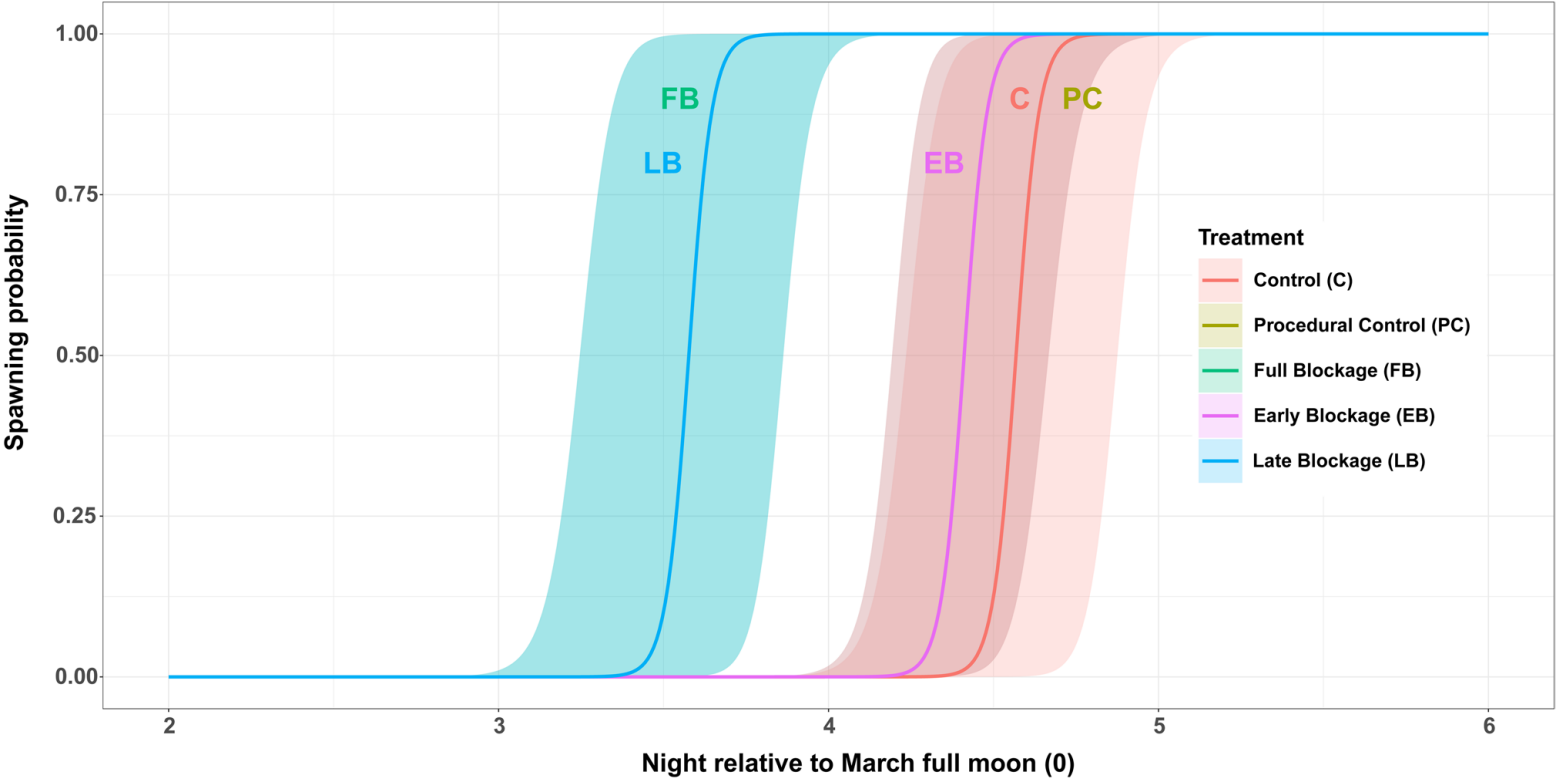
Probability of spawning for different treatments shown as nights relative to full moon night in March. Lines represent probability of spawning based on GLMM models; polygons represent 95% confidence intervals

There was a significant effect of lunar day on spawning, such that all fragments spawned after the full moon (GLMM, *P* < 0.001, *R*^2^ = 0.7). While treatments Full Blockage (FB) and Late Blockage (LB) induced spawning one night earlier than control treatments (GLMM, *P* < 0.001, *R*^2^ = 0.7, Tukey HSD test; *P* < 0.001), this effect was absent for treatment Early Blockage (EB). Our models showed that those treatments that provided an artificial period of darkness post-sunset had a positive effect on spawning timing, taking place before the controls (Fig. 3).

### Effect of Moonlight blocking treatments, April spawning

**Fig. 4:**
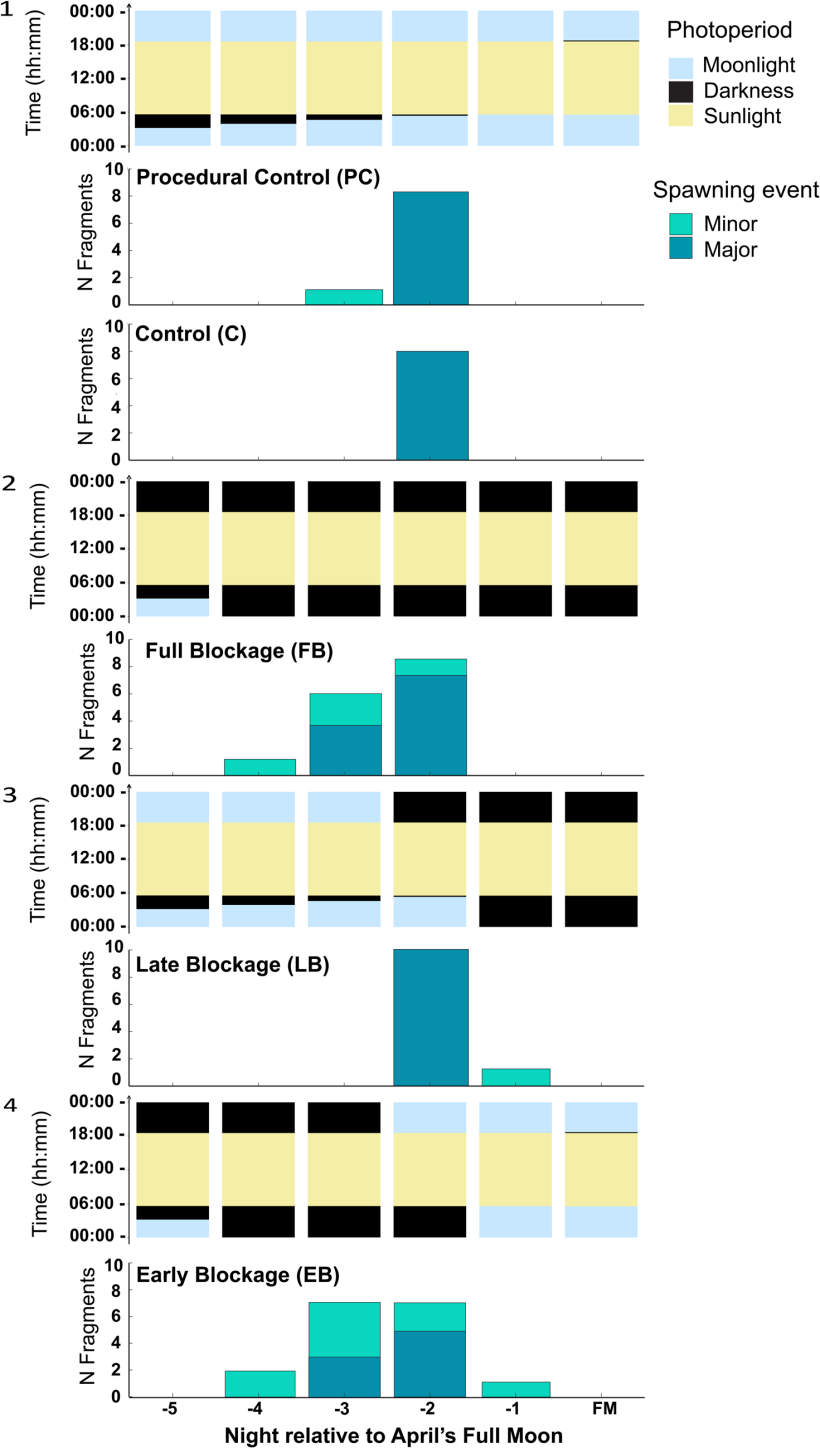
Spawning of *A.* aff. *hyacinthus* colony fragments in the April experiment. Panels 1–4 illustrate the daily light-darkness profiles corresponding to each treatment. For each day, yellow, pale blue, and black bars in the light regime panels indicate periods of sunlight, moonlight, and darkness, respectively, experienced by the colony fragments in each treatment. (1) Environmental conditions, treatments Control (C), and Procedural Control (PC), based in sun and moon rise and set times in Koror, in which no moonlight-darkness manipulations were performed. (2) Full Blockage (FB) treatment, moonlight was blocked post-sunset from 5 days prior to the full moon day until the end of experiment. (3) Late Blockage (LB) treatment, coral fragments were exposed to environmental moonlight conditions until 2 days prior FM day then given total darkness post-sunset from the day after full moon. (4) Early Blockage (EB) treatment, corals were exposed to total darkness post-sunset for the first three nights then ambient environmental conditions until the end of the experiment. Bar graphs underneath light regime panels indicate the spawning pattern on each treatment. Dark teal bars indicate major spawning, light teal bars indicate minor spawning

In April, most fragments (~ 90%) also underwent major spawning with main night of spawning showing variation within treatments (Fig. 4). Three fragments displayed only minor spawning, four fragments died due to *Drupella* spp. predation, of these four, one had a major spawning event, one had only a minor spawning event before dying, and two fragments did not spawn. During one of the daily samplings, it was found that one corner of the platform was not sufficiently raised above the substrate, allowing *Drupella* spp. snails to reach the coral fragments. Once the platform was fixed and snails removed, there was no further mortality. All the Control and Procedural Control fragments spawned two nights before FM. Some fragments in the Full Blockage treatment also spawned earlier than controls, with four fragments spawning three nights prior to FM. However, seven fragments in the Full Blockage treatment spawned two nights before FM (one fragment had two major spawning events). Within the Early Blockage treatment, three fragments spawned three nights before FM while five fragments spawned the following night, including a fragment that also spawned the previous night. All fragments on the Late Blockage treatment spawned on the same night as the control fragments.

**Fig. 5:**
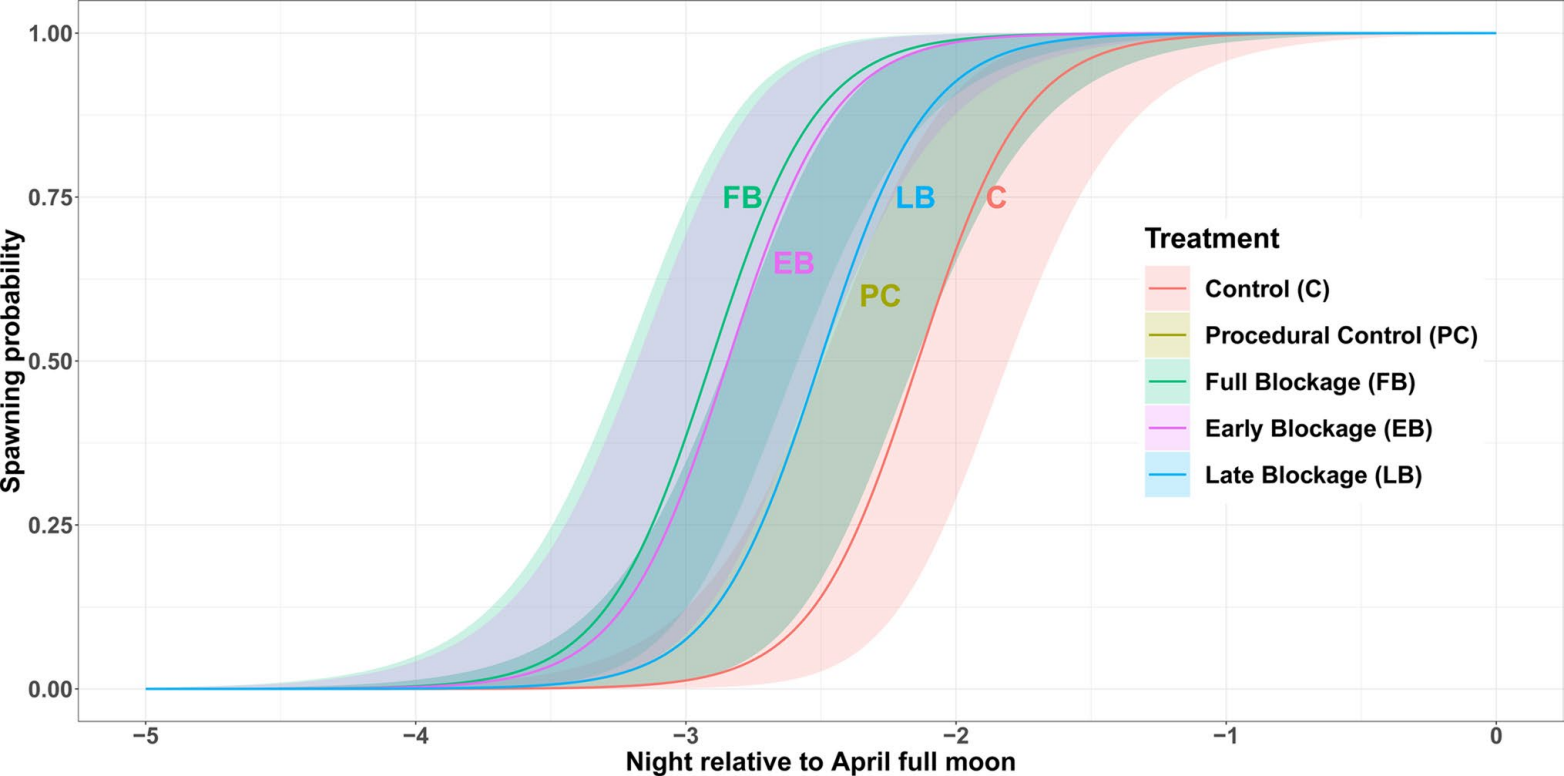
Probability of spawning for different treatments shown as nights relative to full moon night in April (0). Lines represent probability of spawning based on GLMM models; polygons represent 95% confidence intervals

Full Blockage (FB) treatment had a significative effect on the night of spawning of *Acropora* aff*. hyacinthus* fragments (GLMM, *P* < 0.001, *R*^2^ = 0.9, Tukey HSD test; *P* < 0.05). Lunar night (night relative to the full moon) was also statistically significant (GLMM, *P* < 0.001, *R*^2^ = 0.9), and there was an effect of Early Blockage (EB) treatment, although with a higher level of uncertainty (GLMM, *P* < 0.1, *R*^2^ = 0.9, Tukey HSD Test; *P* < 0.05). Models predicted again an earlier probability of spawning in fragments that had been given a darkness cue compared to the controls (Fig. 5). For full model reports and post hoc tests, see Tables S5 and S6.

## Discussion

Previous research suggested that moonlight is key for maintaining rhythmicity of coral spawning (Ayalon et al. 2021; Kaniewska et al. 2015; Rosenberg et al. 2017; Wuitchik et al. 2019). However, the exact processes by which lunar and diel rhythmicity influence spawning synchrony are still unknown. Here, we document spawning of *Acropora* aff*. hyacinthus* corals in Palau under a set of moonlight-darkness experimental conditions. Spawning occurred in March and April, reflecting the split spawning season for *Acropora* on Palauan reefs in 2022. Our results show a variation in the lunar night (and phase) of spawning between months as control fragments spawned 5 days after the March full moon and 2 days before the April full moon. Research to date has suggested that moonlight plays a key role as a cue for the synchronized behaviour of coral spawning (Boch et al. 2011; Kaniewska et al. 2015; Oldach et al. 2017; Rosenberg et al. 2017; Brady et al. 2016). However, here, we show that blocking moonlight near full moon night retained spawning synchronicity in March and to a lesser extent in April and advanced night of spawning compared to natural rhythms during both months. Our findings taken together suggest that a post-sunset darkness period also has a key role to play in spawning timing in an *Acropora* coral.

We documented differences in the response to the onset of darkness post-sunset between March and April spawning experiments. Under ambient environmental conditions, Control and Procedural Control fragments spawned predominantly on the fifth night after the March full moon (Fig. 2), consistent with the expected spawning window for *Acropora* in Palau. At the time of spawning, these fragments had experienced a natural period of darkness post-sunset of at least 1 h per night prior to moonrise for 3 consecutive nights. In contrast, fragments in the Full Blockage and Late Blockage treatments in March advanced their spawning by one night relative to controls. This indicates a response to being exposed to full nights of post-sunset darkness prior to spawning. Full Blockage fragments were exposed to a total of 6 nights of full darkness after sunset; however, these spawned the same night as Late Blockage fragments (which had only been exposed to 3 nights of full darkness), suggesting that fragments could be susceptible to the darkness cue only during a specific time window. This could be related to the final stages of gamete maturation, such as germinal vesicle migration and breakdown (Suwa and Nakamura 2018; Lai et al. 2022). Moreover, the spawning pattern of fragments in the Early Blockage treatment in March was similar to the controls despite having been exposed to three nights of full darkness at the beginning of the experiment, suggesting fragments were not sensitive to the cue at that point.

Under ambient conditions, corals would get a darkness period post-sunset of at least 1 h from the 2nd night after the full moon. We hypothesize that there is a cumulative effect of darkness post-sunset on spawning timing and that at least 2–3 nights with a minimum of 1 h of post-sunset darkness are needed to induce spawning. Interestingly, fragments in the Early Blockage treatment received the moonlight cue after experiencing the darkness cue during the previous nights. That sequence adjusted their spawning timing to align with the normal pattern observed in control treatments. There are three possible explanations for this: (1) moonlight may have modulated or reset an internal circalunidian clock, as shown for other marine invertebrates (Zurl et al. 2022); (2) moonlight may have a suppressive effect on spawning, as demonstrated by Lin et al. (2021); (3) a series of consecutive light-darkness cues (e.g., sunlight followed by progressively longer periods of darkness, then moonlight) can influence spawning (Brady et al. 2016; Rosenberg et al. 2017; Lin et al. 2021).

The patterns that emerged from the April spawning were different than those of the March spawning. In April, corals in all treatments spawned no later than 2 days prior to the full moon, when there was no natural darkness post-sunset period, as the moon had already risen before sunset. This spawning pattern could indicate a disconnection between lunar cues and other environmental cues to allow corals to spawn amid favourable environmental conditions, such as within a certain range of SST (Foster et al. 2018, Lin & Nozawa 2023), overriding the natural darkness cue the consecutive spawning month during split spawning years (Foster et al. 2018). Although the natural darkness cue was overridden or not necessary in April spawning, fragments in the Full and Early Blockage treatments advanced their spawning with respect to the controls (Fig. 4). This finding reinforces the theory that a period of post-sunset darkness can influence the timing of spawning.

Studies testing the effect of the moonlight-darkness interplay on *Acropora* corals are very scarce, and to our knowledge, experimental tests had not been carried out in situ until now. Boch et al. (2011) tested the effect of different light spectra on spawning synchrony finding that mean spawning night advanced when light was blocked. However, they studied a different species, *Acropora humilis*, in an ex situ experiment and peak spawning night of their darkness treatment coincided with that of their other treatments. Kaniewska et al. (2015) investigated the effect of different ex situ light treatments on *Acropora millepora* and reported that colonies kept in darkness after sunset did not spawn at all. However, our results suggest that a period of darkness is not only necessary to advance or trigger spawning, but the day relative to the full moon in which periods of darkness occur is also relevant. These contrasting differences with our findings could be explained by reasons such as species-specific responses to light-darkness cycles, methodological differences, or colony stress effects. Variations in spawning timing due to artefact effects, stress in transport, and relocation of coral fragments are possible. In our experiments, although the environmental conditions in the host reef might vary from those of the donor reef, e.g., water flow, turbidity, and SST, these would have impacted all treatments equally. Artefact effects were ruled out given the fact that in both experiments controls and procedural controls spawned at the same time.

Research investigating the environmental cues responsible for spawning timing and synchrony in coral families other than *Acropora* are rare, although a growing number of experiments addressing the effect of lunar photoperiod, artificial light, and differences in spawning time or gene expression are starting to emerge. Lin and colleagues found that *D. speciosa* fragments consistently spawned 4 days after being exposed to total darkness after sunset, irrespective of the starting date relative to the full moon. These findings match our finding that a prolonged period of darkness after sunset advanced spawning night irrespectively if given before (April) or after (March) the full moon. Extended periods of night illumination during nights close to FM are needed to fine tune swarming and reproduction timing in bristle worms to the darkest times at night (Zurl et al. 2022). Molecular evidence suggests that bristle worms present a moonlight entrained monthly oscillator able to discern lunar phase using cryptochromes (L-Cry)—similar to those present in corals—that allow them to track moonlight duration (Poehn et al. 2022). If a similar mechanism could apply to corals, then masking moonlight around full moon day could have an impact in spawning, which may be another explanation for the differences in spawning timing between fragments given the darkness cue *versus* controls. However, this would not explain why EB fragments in March spawned when they did. When we blocked moonlight for three nights, and then exposed them to natural moonlight conditions, spawning was not statistically different from controls, suggesting the artificial spawning cue can be reset or disrupted, or that fragments were not sensitive to the cue at that point in the cycle (Fig. 2, treatment EB).

The exact mechanisms in which lunar phase and light modulate coral spawning are still elusive. Recent research has shown that gene and protein expression in scleractinian corals vary over the lunar cycle due to natural variations in moonlight (Brady et al. 2016; Oldach et al. 2017). Thus, artificially induced dark or lit periods such as those derived from variations in cloud cover or light pollution can disrupt circalunar clocks by masking entraining cues. Moreover, the interactive effect produced by seasonal changes in temperature; lunar and diurnal cycles in the expression of developmental genes in *Acropora* corals (Wuitchik et al. 2019) indicate the complex and intricate regulation of biological processes, such as spawning time, by an array of endogenous and exogenous cues. Recent evidence has shown that warmer SST correlates with earlier spawning nights relative to the full moon, and an earlier time of spawning after sunset, both in *Acropora* and merulinid corals (Lin and Nozawa 2023). Further, a recently proposed coincidence model hypothesized that spawning in *Acropora* corals was best explained by a combination of moonlight signals and seawater temperature. Rapid seasonal temperature cues would induce gamete maturation, while the ultimate spawning night and timing would be determined by the presence or absence of moonlight over a specific time window of sensitivity (Komoto et al. 2023). However, empirical tests such as those described in this study are needed to determine the windows of sensitivity of different coral taxa to these exogenous cues.

The results presented here provide further insight into the effect of exogenous cues on coral reproductive phenology. We demonstrate that *Acropora* aff*. hyacinthus* corals can spawn synchronously in the absence of moonlight during the week leading to spawning. We further demonstrate that a period of darkness post-sunset a few days prior to spawning can be a proximate cue for an *Acropora* coral. We also document different lunar nights of spawning within corals from the same assemblage during a split spawning year. Future research is needed to disentangle the effect of temperature, moonlight, and darkness regimes in the spawning of different coral taxa. Experimental tests in controlled environments, such as mesocosm systems, could resolve and separate the effect of moonlight and darkness cues in coral spawning timing. Further manipulative experiments and long spawning monitoring programs using standardized methods are required to better understand the extent to which the reproductive phenology of scleractinian corals is influenced by exogenous cues. Particularly, identifying the windows of sensitivity to each cue and their role in spawning synchrony at three main levels, month, day, and time of spawning, is key to predict and buffer the effect of anthropic disturbances on coral spawning synchrony.

## Supplementary Information

Below is the link to the electronic supplementary material.

Supplementary file 1 (PDF 4305 KB)

Supplementary file 2 (PDF 4073 KB)

Supplementary file 3 (DOCX 17 KB)

Supplementary file 4 (PDF 4447 KB)

Supplementary file 5 (PDF 192 KB)

## Data Availability

All original datasets and code (R version 4.2.2) generated during and/or analysed during the current study are available from the corresponding author on reasonable request.
