# Supplementary material for: Evaluating the role of moonlight-darkness dynamics as proximate spawning cues in an Acropora coral
Source: Coral Reefs. 2025 Jan 28;44(2):501–12. doi: 10.1007/s00338-025-02618-9 (PMC11950126; doi:10.1007/s00338-025-02618-9)

## A fragment from each *Acropora* aff. *hyacinthus* colony (March)

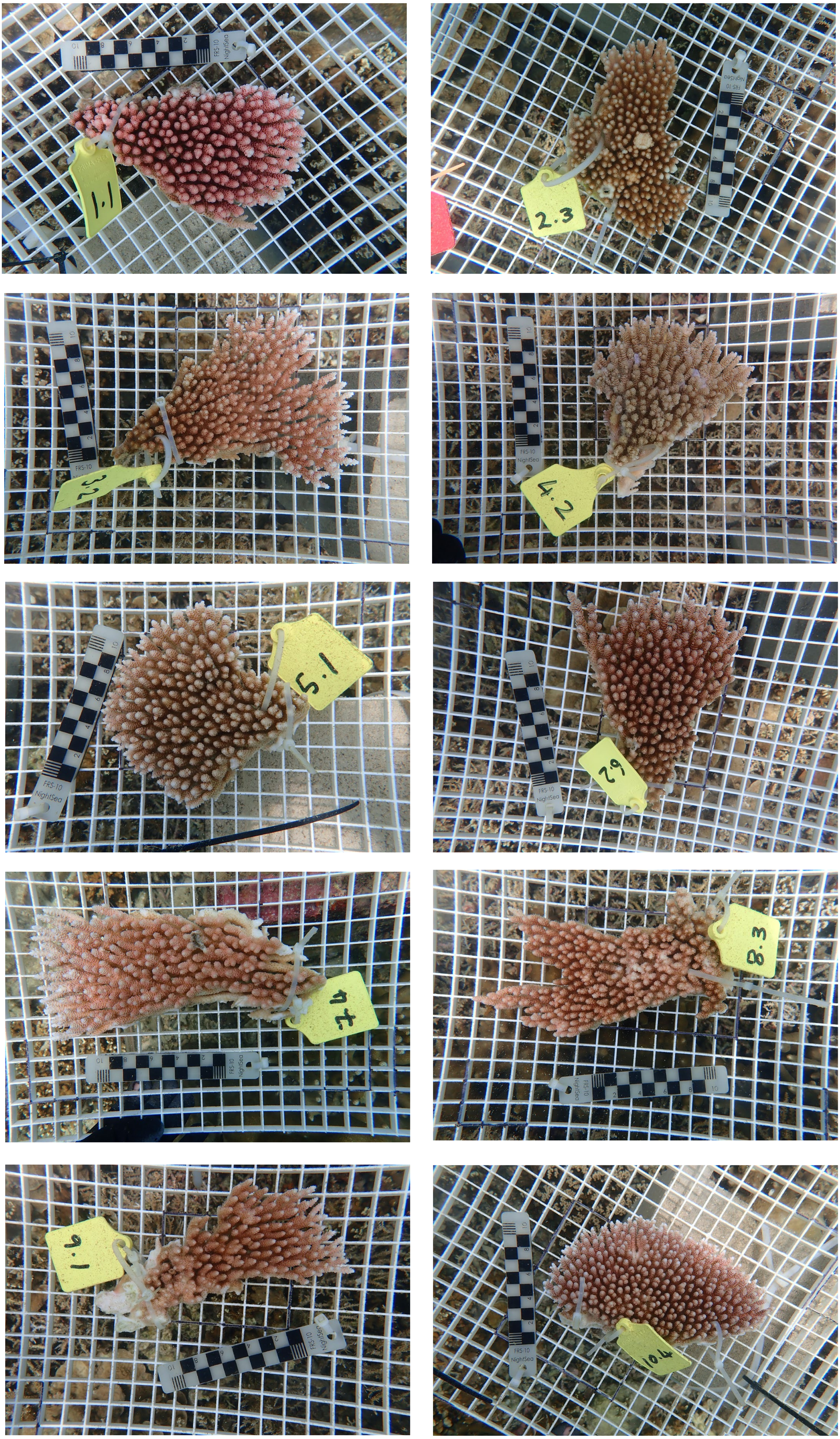

A fragment from each *Acropora* aff. *hyacinthus* colony (April)

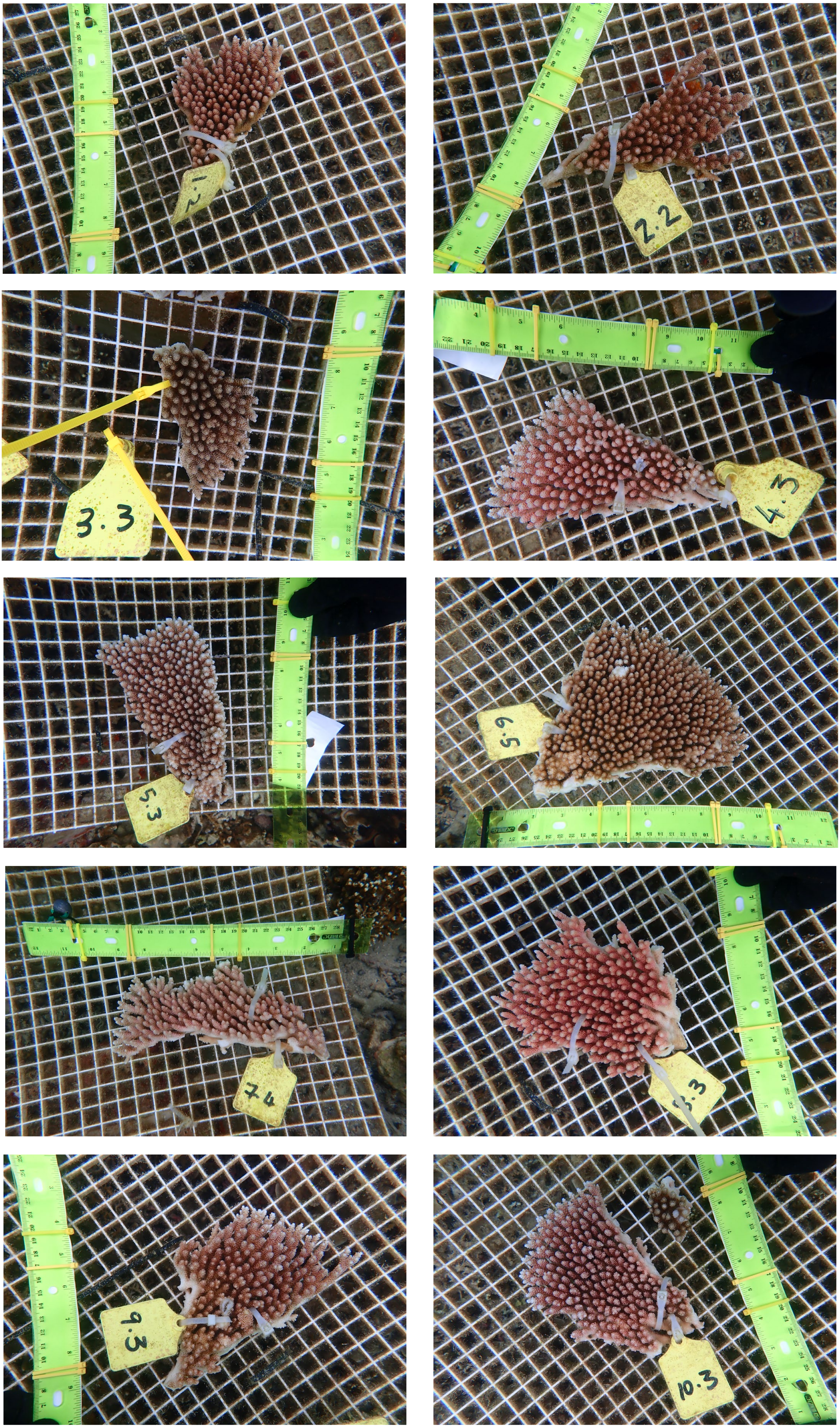

Supplement: Supplementary file 1 — (PDF 4305 KB) [file 338_2025_2618_MOESM1_ESM.pdf]
