# Supplementary figures and images for: Evaluating the role of moonlight-darkness dynamics as proximate spawning cues in an Acropora coral
Source: Coral Reefs. 2025 Jan 28;44(2):501–12. doi: 10.1007/s00338-025-02618-9 (PMC11950126; doi:10.1007/s00338-025-02618-9)

# Daily average SST experimental site vs Double Reef March-April 2022

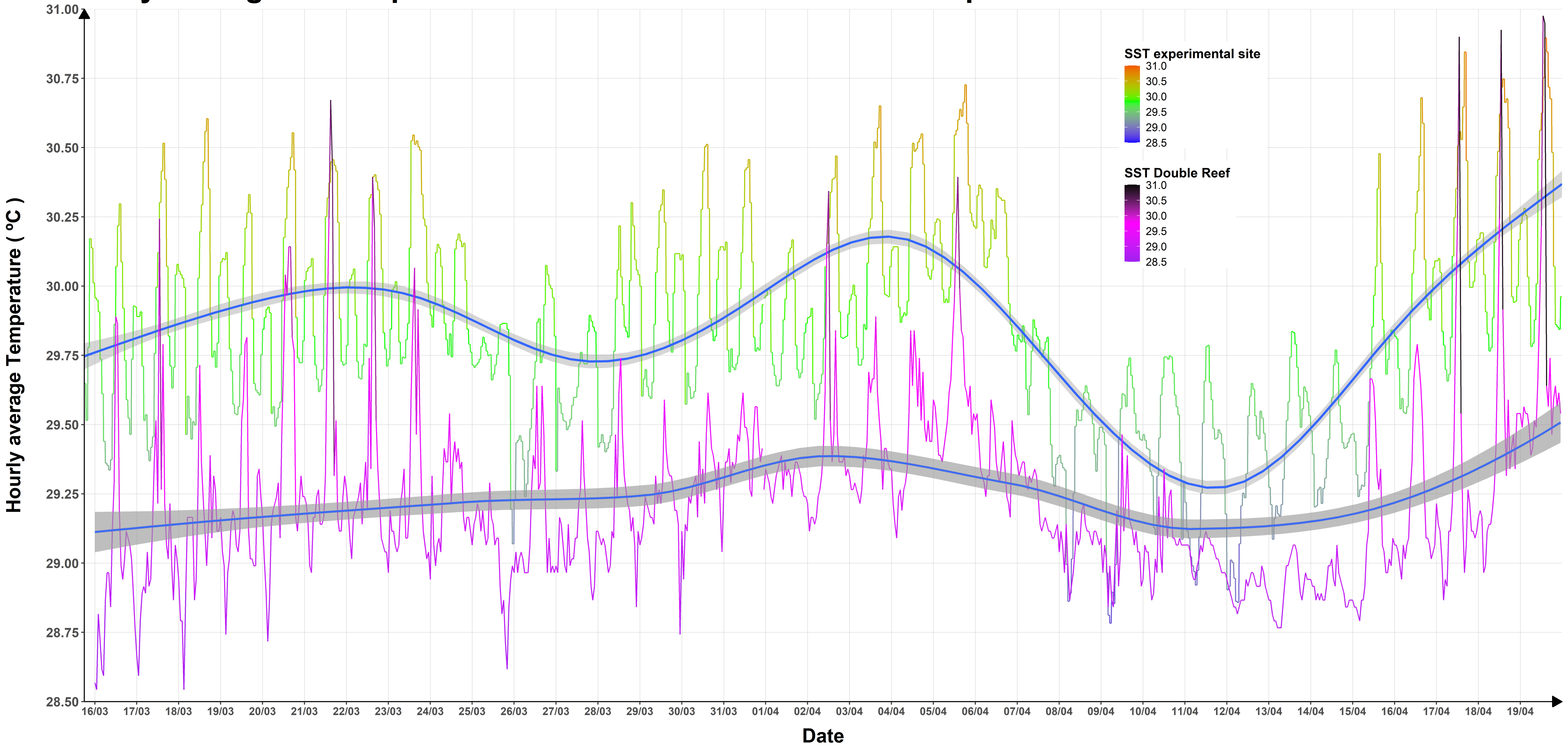

Supplement: Supplementary file 2 — (PDF 4073 KB) [file 338_2025_2618_MOESM2_ESM.pdf]

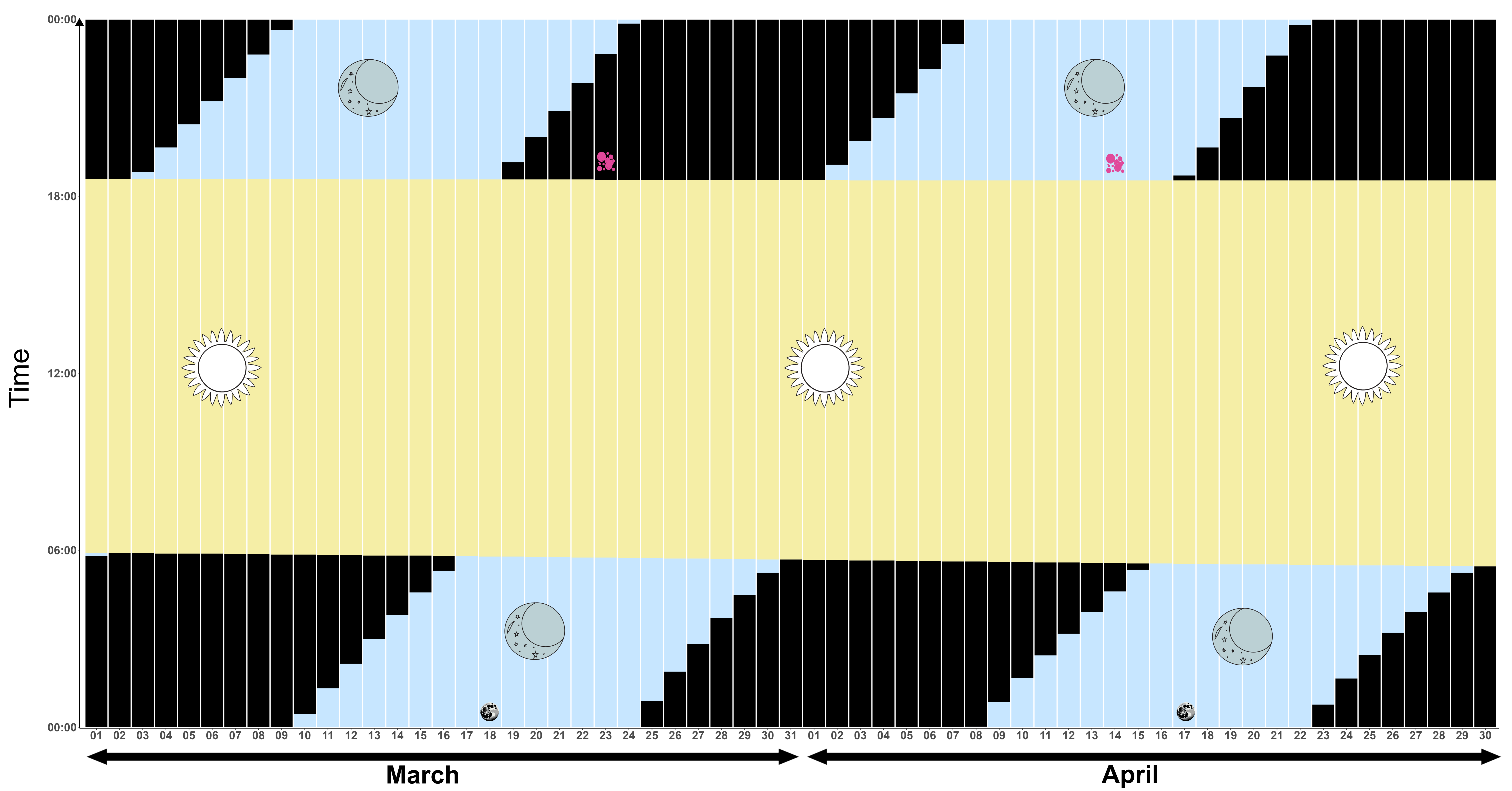

Supplement: Supplementary file 4 — (PDF 4447 KB) [file 338_2025_2618_MOESM4_ESM.pdf]
