# Supplementary material for: Evaluating the role of moonlight-darkness dynamics as proximate spawning cues in an Acropora coral
Source: Coral Reefs. 2025 Jan 28;44(2):501–12. doi: 10.1007/s00338-025-02618-9 (PMC11950126; doi:10.1007/s00338-025-02618-9)
Supplement: Supplementary file 3 — (DOCX 17 KB) [file 338_2025_2618_MOESM3_ESM.docx]

Supplementary materials S3.

Table S1. Concept of the experimental design used in March and April experiments.

| **March / April spawning experiments design** | | | | | | | |
| --- | --- | --- | --- | --- | --- | --- | --- |
|  | A = Opaque cover, B = Transparent cover, C = Control (collection cup only) | | | | | | |
| **Treatment** | **Day 1** | **Day 2** | **Day 3** | **Day 4** | **Day 5** | **Day 6** | **Day 7 +** |
| Full blockage | A | A | A | A | A | A | A |
| Early blockage | A | A | A | B | B | B | B |
| Late blockage | B | B | B | A | A | A | A |
| Procedural control | B | B | B | B | B | B | B |
| Control | C | C | C | C | C | C | C |

Table S2. Concept of the random stratified design used in March and April experiments to assign experimental fragments (F_ID = Colony.Fragment, e.g., F_ID 1.1 = Colony 1, fragment 1) to their respective treatments (Treatment; PC = Procedural Control, FB = Full Blockage, LB = Late Blockage, EB = Early Blockage, C = Control).

| F_ID | Treatment | F_ID | Treatment | F_ID | Treatment | F_ID | Treatment | F_ID | Treatment |
| --- | --- | --- | --- | --- | --- | --- | --- | --- | --- |
| 1.1 | PC | 3.1 | LB | 5.1 | C | 7.1 | FB | 9.1 | EB |
| 1.2 | FB | 3.2 | EB | 5.2 | PC | 7.2 | LB | 9.2 | C |
| 1.3 | LB | 3.3 | C | 5.3 | FB | 7.3 | EB | 9.3 | PC |
| 1.4 | EB | 3.4 | PC | 5.4 | LB | 7.4 | C | 9.4 | FB |
| 1.5 | C | 3.5 | FB | 5.5 | EB | 7.5 | PC | 9.5 | LB |
| 2.1 | FB | 4.1 | EB | 6.1 | PC | 8.1 | LB | 10.1 | C |
| 2.2 | LB | 4.2 | C | 6.2 | FB | 8.2 | EB | 10.2 | PC |
| 2.3 | EB | 4.3 | PC | 6.3 | LB | 8.3 | C | 10.3 | FB |
| 2.4 | C | 4.4 | FB | 6.4 | EB | 8.4 | PC | 10.4 | LB |
| 2.5 | PC | 4.5 | LB | 6.5 | C | 8.5 | FB | 10.5 | EB |
