# Supplementary material for: Evaluating the role of moonlight-darkness dynamics as proximate spawning cues in an Acropora coral
Source: Coral Reefs. 2025 Jan 28;44(2):501–12. doi: 10.1007/s00338-025-02618-9 (PMC11950126; doi:10.1007/s00338-025-02618-9)
Supplement: Supplementary file 5 — (PDF 192 KB) [file 338_2025_2618_MOESM5_ESM.pdf]

Table S5. Effect of experimental treatments spawning probability of *Acropora* aff. *hyacinthus.* Estimate, standard error (Std. Error), z-score (z value) and *p-value* obtained from GLMMs. Significance level: *** p < 0.001; ** p < 0.01; ** p < 0.05. SNRFM refers to night of spawning in the lunar cycle relative to the full moon. Pairwise comparisons obtained from post-hoc tests, Estimated Marginal Means (EMMs) and pairwise comparisons Tukey's Honest Significant Difference (HSD) test, significance level (pairwise): ns= non-significant.

**March GlmmTMB model output**					
Factor	Estimate	Std. Error	z value	*p*	pairwise
SNRFM	28.49	7.26	3.93	***	
Treatment PC	4.48	4.54	0.99		ns
Treatment FB	28.25	8.60	3.28	**	ns
Treatment LB	28.25	8.60	3.28	**	ns
Treatment EB	4.48	4.54	0.99		ns
R2 = 0.7 AIC = 44.5					

**April GlmmTMB model output**					
Factor	Estimate	Std. Error	z value	*p*	pairwise
SNRFM	5.04	0.98	5.12	***	
Treatment PC	1.83	1.14	1.61		ns
Treatment FB	3.87	1.29	3.00	**	ns
Treatment LB	1.83	1.14	1.61		ns
Treatment EB	3.55	1.29	2.76	**	ns
R2 = 0.9 AIC = 84.0					

Table S6: Tukey's HSD test results for pairwise comparisons of treatment groups based on generalized linear mixed models (GLMMs). Degrees of freedom (df) are treated as infinite because the model uses maximum likelihood estimation, which assumes an asymptotic framework. The results include the estimated differences in response, standard errors (SE), and p-values adjusted for multiple comparisons.

**March**					
Contrast	Estimate	SE	df	z.ratio	p.value
C - PC	-4.48	4.544	Inf	-0.986	0.862
C - FD	-28.251	8.602	Inf	-3.284	0.009
C - CD	-28.251	8.602	Inf	-3.284	0.009
C - SD	-4.48	4.544	Inf	-0.986	0.862
PC - FD	-23.772	6.629	Inf	-3.586	0.003
PC - ED	-23.772	6.629	Inf	-3.586	0.003
PC - SD	0	2.508	Inf	0	1
FD - ED	0	4.718	Inf	0	1
FD - SD	23.772	6.629	Inf	3.586	0.003
ED - SD	23.772	6.629	Inf	3.586	0.003
**April**					
Contrast	Estimate	SE	df	z.ratio	p.value
C - PC	-1.831	1.139	Inf	-1.608	0.492
C - FD	-3.867	1.29	Inf	-2.998	0.023
C - ED	-1.832	1.139	Inf	-1.609	0.492
C - SD	-3.551	1.287	Inf	-2.759	0.046
PC - FD	-2.036	1.086	Inf	-1.874	0.331
PC - ED	0	1.068	Inf	0	1
PC - SD	-1.72	1.102	Inf	-1.561	0.522
FD - ED	2.035	1.086	Inf	1.873	0.332
FD - SD	0.316	0.994	Inf	0.318	0.998
ED - SD	-1.719	1.102	Inf	-1.561	0.523
